# Venom-Induced Recurrent Thrombocytopenia: A Model of Intervention-Driven Platelet Modulation

**DOI:** 10.3390/toxins17120605

**Published:** 2025-12-17

**Authors:** Mojca Dobaja Borak, Katarina Reberšek, Tihana Kurtović, Adrijana Leonardi, Igor Križaj, Miran Brvar

**Affiliations:** 1 Centre for Clinical Toxicology and Pharmacology, University Medical Centre Ljubljana, 1000 Ljubljana, Slovenia; mojca.dobaja@kclj.si; 2 Doctoral School, Faculty of Medicine, University of Ljubljana,1000 Ljubljana, Slovenia; 3 Department of Haematology, University Medical Centre Ljubljana, 1000 Ljubljana, Slovenia; katarina.rebersek@kclj.si; 4 Centre for Research and Knowledge Transfer in Biotechnology, University of Zagreb, 10000 Zagreb, Croatia; tihana.kurtovic@unizg.hr; 5 Department of Molecular and Biomedical Sciences, Jožef Stefan Institute, 1000 Ljubljana, Slovenia; adrijana.leonardi@ijs.si (A.L.); igor.krizaj@ijs.si (I.K.); 6 Center for Clinical Physiology, Faculty of Medicine, University of Ljubljana, 1000 Ljubljana, Slovenia

**Keywords:** *Vipera ammodytes ammodytes*, venom, thrombocytopenia, functional platelets, snaclec

## Abstract

We present the case of a *Vipera ammodytes ammodytes* (*Vaa*, nose-horned viper)-bitten patient with recurrent thrombocytopenia. A 53-year-old patient envenomated by *Vaa* experienced three episodes of venom-dependent thrombocytopenia (4, 57 and 11 × 10^9^/L), all of which we managed with antivenom Fab fragments. Despite these three severe episodes of thrombocytopenia within 24 h, platelet function remained intact, as demonstrated by normal thromboelastometry and aggregometry (96, 126, and 150 U) results after antivenom was administered and the platelet count normalized. Furthermore, flow cytometry showed only 0.3–1.7% expression of P-selectin on platelets, indicating that platelets did not activate but remained functional during and after thrombocytopenia. We assessed platelet function using rotational thromboelastometry, which evaluates the overall kinetics of hemostasis, including clot formation and stability. We performed aggregometry, which also reflects platelet function, only when the platelet count was within the normal range. Flow cytometry quantified P-selectin expression as a key marker of platelet activation. This case demonstrates that a component of *Vaa* venom can repeatedly induce venom-dependent thrombocytopenia, which is reversible by intervention, while platelet function remains intact.

## 1. Introduction

In Europe, *Vipera ammodytes ammodytes* (*Vaa*, nose-horned viper) is the most dangerous of the European vipers due to its large size (up to 95 cm), long fangs (up to 13 mm) and high venom toxicity induced by hematotoxic and neurotoxic components [1,2,3,4]. *Vaa* venom contains metalloproteinases and their tripeptide inhibitors, secreted phospholipases A_2_, serine proteases, disintegrins, C-type lectin-like proteins, L-amino acid oxidases, cysteine-rich secretory proteins, Kunitz-type protease inhibitors, and vascular endothelial growth factors [5,6,7].

The most common laboratory findings in *Vaa* envenomation are mild leukocytosis and severe thrombocytopenia [8]. Severe thrombocytopenia is an indication for antivenom therapy in *Vaa* envenomation, alongside progression of edema beyond a major joint (reaching the arm or thigh) and the presence of systemic signs.

The antivenom ViperaTAb^®^ (MicroPharm Ltd., Newcastle Emlyn, UK), a monospecific ovine Fab preparation targeting *Vipera berus berus* (*Vbb*) venom, may be used when a *Vaa*-specific antivenom is unavailable [9]. However, its paraspecific efficacy in *Vaa* bites may be limited. Recent proteomic studies show that *Vbb* venom is much less complex than *Vaa* venom [8]. In particular, it contains lower levels of snake C-type lectin-like proteins (snaclecs) and lacks ammodytoxins, the neurotoxic secreted phospholipases A_2_ responsible for the characteristic neurotoxic signs of *Vaa* envenoming, while snaclecs are likely responsible for venom-induced thrombocytopenia [8]. Because of these compositional differences, patients may require additional doses of paraspecific antivenom depending on the progression of envenomation (e.g., further spread of local signs or worsening laboratory findings, particularly thrombocytopenia) [9]. Nevertheless, ViperaTAb demonstrates substantial cross-reactivity with *Vaa* venom in vitro, and in vivo studies show that it reduces *Vaa*-induced lethality with potency exceeding the minimum requirements of both the British and European Pharmacopoeia [10,11].

The absence of clinically significant bleeding in *Vaa*-envenomed patients suggests that platelet function is preserved despite severe thrombocytopenia [8].

In a *Vaa*-envenomed patient with thrombocytopenia, we measured serum levels of venom by ELISA [8] and assessed platelet function using rotational thromboelastometry (ROTEM), which evaluates the overall kinetics of hemostasis (clot formation and stability), and aggregometry, which reflects platelet function when the platelet count is normal [12]. Additionally, flow cytometry was employed to measure P-selectin expression, a key marker of platelet activation [13,14].

In this case report, we present a *Vaa*-envenomed patient who experienced three episodes of severe thrombocytopenia, effectively controlled with antivenom Fab fragments while preserving intact platelet function. This demonstrates that the count of functional platelets can be therapeutically modulated, potentially paving the way for novel treatment strategies and drug development.

## 2. Case

A 53-year-old female, with no relevant past medical history or ongoing pharmacotherapy, was bitten by a *Vaa*, resulting in rapid leg swelling and increasing pain. A relative subsequently killed the snake at the site, which enabled photography and identification.

Four hours post-bite, she presented with vomiting, diarrhea, and hypotension (80/60 mmHg). Laboratory results 4 h post-bite showed profound thrombocytopenia (4 × 10^9^/L) and *Vaa* venom level of 67 ng/mL (Table 1). The patient was classified as grade 2b on the modified Audebert clinical severity scale [15]. As *Vaa*-specific antivenom was unavailable, we promptly administered a monospecific ovine Fab antivenom raised against whole *Vbb* venom (ViperaTAb^®^, 8 mL in 100 mL 0.9% NaCl over 30 min) within 1 h due to the severe thrombocytopenia. The treatment reduced venom levels to 30.5 ng/mL and restored the platelet count to 183 × 10^9^/L. ROTEM (Pentapharm, Munich, Germany) showed normal clot formation time (CFT) for EXTEM and INTEM (115 and 103 s) and maximum clot firmness (MCF) for EXTEM and INTEM (56 and 58 mm), indicating preserved platelet function. The aggregometry TRAPtest performed using the Multiplate^®^ analyzer (Roche Diagnostics, Mannheim, Germany) was also normal (96 U), confirming adequate platelet function. Flow cytometry (Navios, Beckman Coulter, Brea, CA, USA) showed that only 1.7% of platelets expressed P-selectin (FITC-labeled monoclonal antibody directed against CD62P; Beckman Coulter, Brea, CA, USA) (Table 1).

Four hours later, 10 h after the bite, thrombocytopenia reoccurred (59 × 10^9^/L) and ROTEM revealed prolonged CFT and decreased MCF, so a second dose of antivenom was administered 11 h after the bite due to recurrent severe thrombocytopenia. The platelet count normalized again (207 × 10^9^/L) and ROTEM results returned to baseline values within 1 h (Table 1).

The next morning, 22 h after the bite, thrombocytopenia reoccurred (11 × 10^9^/L), and the venom level was again high (72 ng/mL). ROTEM showed prolonged CFT (798 and 995 s) and low MCF (27 and 25 mm) for EXTEM and INTEM, while flow cytometry indicated only 0.7% platelet activation. The third dose of antivenom was given 23 h post-bite for severe thrombocytopenia, leading to platelet count normalization within 1 h (196 × 10^9^/L) and a reduction in venom level to 34 ng/mL. ROTEM results also normalized, with CFT (86 and 86 s) and MCF (60 and 57 mm) for EXTEM and INTEM, respectively. The aggregometric TRAPtest was within normal limits (150 U), and flow cytometry showed only 0.3% platelet activation (Table 1). ROTEM showed normal MCF (9–14 mm) for FIBTEM (Table 1).

Platelet counts were determined using the impedance method on Sysmex hematology analyzer XN-2000 (Sysmex Corporation, Kobe, Japan) as part of the complete blood count, and were confirmed by flow cytometry using fluorescent dyes that bind to platelet RNA (Sysmex Corporation, Kobe, Japan). Pseudo-thrombocytopenia or analytical error due to possible in vitro formation of aggregates within a tube was ruled out by microscopic examination of the blood smear and the use of different anticoagulants. The microscopic examination of the blood smear showed no aggregates or schistocytes. Direct and indirect anti-platelet antibody tests were negative, as were direct and indirect Coombs tests.

The patient exhibited mild coagulopathy, with D-dimer levels markedly elevated to 35,000 µg/L (reference level < 500 µg/L). However, she did not develop venom-induced consumptive coagulopathy (VICC), as her lowest prothrombin time ratio was 0.54 s (reference 0.7–1.3), while activated partial thromboplastin time and fibrinogen remained within the normal ranges (Table 1). Throughout hospitalization, no bleeding or rhabdomyolysis was observed, as creatine kinase (3.3 µkat/L; reference < 2.41 µkat/L) and myoglobin levels (156 µg/L; reference < 106 µg/L) were only slightly elevated (Table 1). Plasma vWF levels were elevated at 6 and 12 h after the bite but returned to normal from the second day onward (Table 1). By the third day after the bite, platelet count and coagulation tests had normalized, leg swelling and pain had subsided, and hospital management was no longer required.

### Quantification of Vaa Venom in Serum Samples

Patient blood samples for venom measurement were collected in serum tubes upon arrival at the emergency department, and before and after administration of ViperaTAb (Table 1). The samples were immediately centrifuged, aliquoted and frozen at −50 °C until venom quantification. Quantification of *Vaa* venom in serum samples was performed by coating the microtiter plate with rabbit anti-*Vaa* venom IgG (5 μg/mL). After the blocking step, 10-fold diluted sera were added, together with whole venom (Institute of Immunology Inc., Croatia), which was used as the standard. It was applied in eight serial 1.5-fold dilutions (starting at 15 ng/mL) prepared in 10% serum from a non-bitten donor. The plate was incubated sequentially with horse anti-*Vaa* venom IgG (5.7 μg/mL) and HRP-anti-equine IgG (0.2 µg/mL) (Sigma-Aldrich, St. Louis, MO, USA) [8]. Rabbit and horse anti-*Vaa* venom IgGs were obtained by immunizing the respective animals with whole venom (Institute of Immunology Inc., Croatia) and purifying the antibodies from the collected sera by affinity chromatography. The substrate was *o*-phenylenediamine (0.6 mg/mL) (Sigma-Aldrich, St. Louis, MO, USA) in 5.5 mM citrate-phosphate buffer, pH 5.0, with 30% (*v*/*v*) H_2_O_2_ (0.5 μg/mL of OPD solution).

## 3. Discussion

This case demonstrates that *Vaa* venom exposure causes venom-dependent, profound, and transient thrombocytopenia with functional platelets after recovery. Platelet function remains intact despite three episodes of severe thrombocytopenia, as demonstrated by normal thromboelastometry and aggregometry results. Additionally, flow cytometry shows only 0.3–1.7% expression of P-selectin on the plasma membrane of platelets, indicating that these cells do not activate, but remain functional during and after thrombocytopenia. The most remarkable finding in this case is the recurrence of severe thrombocytopenia within 24 h, which is successfully managed with Fab fragments-based antivenom intervention, while preserving platelet function.

The most likely mechanism of thrombocytopenia is platelet agglutination mediated by *Vaa*-snaclec-3/2 in *Vaa* venom, as shown in ex vivo and in vivo studies [16]. *Vaa*-snaclec-3/2 isolated from the crude *Vaa* venom has been shown to induce platelet agglutination and consequently thrombocytopenia by binding to the GPIb platelet receptor [16]. This is supported by ex vivo optical microscopy, which revealed platelet agglutinates in whole blood after exposure to *Vaa*-snaclec-3/2, and *Vaa*-snaclec-3/2 exerted an inhibitory effect on ristocetin-induced platelet agglutination, consistent with its interaction with the GPIb receptor. The latter was further confirmed by flow cytometry using fluorescently conjugated GP-specific antibodies [16]. *Vaa*-snaclec-3/2 is an acidic, non-glycosylated covalent heterodimer consisting of *Vaa-*snaclec-3 (α-subunit of 18 kDa) and *Vaa*-snaclec-2 (β-subunit of 15 kDa) [16]. The kinetics of platelet agglutinates and their possible sequestration site have not yet been studied in detail [17], but pulmonary vein sequestration seems most likely.

Reversible inhibition of platelet activation during thrombocytopenia by an unknown venom component could not be ruled out, given the limitations of assessing platelet function with aggregometry. However, the absence of inhibitors of platelet activation in *Vaa* venom proteome studies supports the conclusion that platelet function was preserved during thrombocytopenia [16].

The recurrence of thrombocytopenic episodes within 24 h reflects a pharmacokinetic mismatch between venom and antivenom, as delayed absorption of venom from peripheral tissues at the bite site continues after Fab-based antivenom is cleared due to its relatively short elimination half-life [8]. Intravenous administration of antivenom containing Fab fragments directed against whole venom rapidly restores platelet counts. These Fab fragments most probably bind the agglutinating venom component (e.g., *Vaa*-snaclec-3/2), promoting dissociation of aggregates into individual, functionally intact platelets that were not activated during agglutination. The released platelets reappear in circulation, thereby resolving thrombocytopenia—albeit only transiently, depending on the relative kinetics of venom and Fab fragments. The brief, transient improvement of thrombocytopenia was also attributable to the administration of only a single dose of paraspecific Fab fragments (8 mL of ViperaTAb). A double dose administered at once is not recommended and has not yet been evaluated. Instead, repeated dosing is advised based on the progression of envenomation, as was done in this case [9,15].

Other possible explanations of the observed transient thrombocytopenia, such as pseudo-thrombocytopenia or consumptive coagulopathy, were excluded. Platelet aggregates as a possible cause of pseudo-thrombocytopenia or an analytical error were not observed in vitro in the test tube. VICC with thrombocytopenia was excluded, as thromboelastometric and aggregometric values were normal after reversal of thrombocytopenia, although elevated D-dimer and a slightly decreased prothrombin time ratio were recorded. No fibrinogen depletion was observed. Furthermore, the very low proportion of P-selectin-expressing platelets (0.3–1.7%) also excluded VICC [18,19]. The possibility of an immunological background of thrombocytopenia was rejected by performing anti-platelet antibody tests. Additional possible causes of thrombocytopenia, such as significant local tissue damage with coagulopathy or sequestration of platelets at the site of envenomation onto the exposed and damaged endothelium, were unlikely, as no consumption of clotting factors and no mechanical damage to red blood cells due to microangiopathic changes leading to schistocyte formation were detected. The patient also had only minimal laboratory signs of rhabdomyolysis. However, local tissue injury at the site of the snakebite most likely caused sufficient endothelial injury and activation to increase plasma vWF levels, a response also observed in other forms of localized vascular injury such as acute coronary syndrome and acute stroke [20], as well as in focal traumatic injuries [21]. The elevated vWF did not originate from platelets because they were not activated.

This case illustrates the potential for rapid, intervention-induced fluctuations in platelet count without compromising function. Despite remarkably swift shifts from just a few platelets to several hundred within 1 h, there is no evidence of platelet activation, inactivation, or bleeding. Such rapid and recurring fluctuations in platelet counts have not been documented in any other known condition or disease. This finding opens up new possibilities for both treatment and research, as induced therapeutic thrombocytopenia could be explored for targeted therapies. For example, in in vivo experiments, *Vaa*-snaclec-3/2 induced thrombocytopenia and protected mice from carotid artery thrombosis induced by ferric chloride, highlighting its potential application in interventional angiology and cardiology [16].

## 4. Conclusions

This case demonstrates that a component of *Vaa* venom can induce recurrent venom-dependent thrombocytopenia, which is reversible with antivenom Fab fragments, while platelet function remains intact. These findings suggest that intervention-induced fluctuations in platelet count are possible and may have diagnostic and therapeutic applications.

## Figures and Tables

**Table 1:** Laboratory results, rotational thromboelastometry (ROTEM), aggregometry assays (TRAPtest) and platelet flow cytometry before and after intravenous administration of Fab antivenom fragments in a *Vaa*-envenomed patient.

Parameter (Unit; Reference Range)										
Time After Bite (h)	4	5	6	10	11	12	22	23	24	48
Venom (ng/mL)	67	Intravenous infusion of 8 mL ViperaTAb diluted in 100 mL 0.9% NaCl, administered over 30 min	30	75	Intravenous infusion of 8 mL ViperaTAb diluted in 100 mL 0.9% NaCl, administered over 30 min	55	72	Intravenous infusion of 8 mL ViperaTAb diluted in 100 mL 0.9% NaCl, administered over 30 min	34	33
White blood count (×10^9^/L; 4.0–10.0)	7.6		20.7	18.2		15.2	13.1		14.1	13.6
Hemoglobin (g/L; 120–150)	142		155	148		138	139		126	117
Platelets (×10^9^/L; 150–410)	4		183	59		207	11		196	168
Sodium (mmol/L; 135–145)	141		139	140		139	141		139	135
Potassium (mmol/L; 3.8–5.5)	3.2		na	4.5		na	5.5		5.0	4.3
Urea (mmol/L; 3.2–7.4)	4.6		4.7	5.8		5.9	4.4		4.3	3.6
Creatinine (µmol/L; 64–107)	87		76	79		77	75		74	72
Creatine kinase (µkat/L; <2.41)	2.6		2.5	na		na	2.9		3.3	3.2
Myoglobin (µg/L; <106)	47		45	48		77	156		138	130
Procalcitonin (µg/L; <0.24)	0.03		0.12	0.75		0.87	0.56		0.35	0.15
Prothrombin time ratio (>0.70)	0.67		0.71	0.65		0.63	0.54		0.61	0.77
International normalized ratio (INR) (<1.30)	1.20		1.16	1.22		1.23	1.34		1.25	1.12
Activated partial thromboplastin time (s; 24.4–36.6)	21.9		na	24.8		na	28.3		26.2	24.5
D-dimer (µg/L; <500)	27,831		35,000	35,000		35,000	26,387		20,108	7307
Fibrinogen (g/L; 1.8–3.5)	2.8		2.49	2.4		2.3	2.4		2.6	2.9
ROTEM										
EXTEM CFT (s; 35–160)	na		115	210		82	798		86	123
EXTEM MCF (mm; 53–72)	na		56	45		60	27		60	55
INTEM CFT (s; 35–110)	na		103	169		87	995		86	101
INTEM MCF (mm; 53–72)	na		58	48		56	25		57	59
FIBTEM MCF (mm; 8-20)	na		11	14		13	9		13	9
TRAPtest (U; 86–159)	na		96	na		126	na		150	151
Platelets with expressed P-selectin (%)	na		1.7	na		na	0.7		0.3	na
vWF-antigen (E/mL; 0.5–1.6)	na		4.56	na		3.73	1.6		1.5	na

Legend: CFT—clot formation time, MCF—maximum clot firmness, TRAP—thrombin receptor activating peptide, na—not available.

## Data Availability

The original contributions presented in this study are included in the article. Further inquiries can be directed to the corresponding author.
